# Paving the way for a better understanding of the pathophysiology of gait impairment in myotonic dystrophy: a pilot study focusing on muscle networks

**DOI:** 10.1186/s12984-019-0590-0

**Published:** 2019-09-18

**Authors:** Antonino Naro, Simona Portaro, Demetrio Milardi, Luana Billeri, Antonino Leo, David Militi, Placido Bramanti, Rocco Salvatore Calabrò

**Affiliations:** 1 IRCCS Centro Neurolesi Bonino Pulejo, via Palermo, SS 113, Ctr. Casazza, 98124 Messina, Italy; 2 Stomatodental Center, Messina, Italy

**Keywords:** Signal synergies, Muscle networks, Myotonic dystrophy type 1, Gait, Time–frequency analysis, Multivariate analysis

## Abstract

**Background:**

A proper rehabilitation program targeting gait is mandatory to maintain the quality of life of patients with Myotonic dystrophy type 1 (DM1). Assuming that gait and balance impairment simply depend on the degree of muscle weakness is potentially misleading. In fact, the involvement of the Central Nervous System (CNS) in DM1 pathophysiology calls into account the deterioration of muscle coordination in gait impairment. Our study aimed at demonstrating the presence and role of muscle connectivity deterioration in patients with DM1 by a CNS perspective by investigating signal synergies using a time-frequency spectral coherence and multivariate analyses on lower limb muscles while walking upright. Further, we sought at determining whether muscle networks were abnormal secondarily to the muscle impairment or primarily to CNS damage (as DM1 is a multi–system disorder also involving the CNS). In other words, muscle network deterioration may depend on a weakening in signal synergies (that express the neural drive to muscles deduced from surface electromyography data).

**Methods:**

Such an innovative approach to estimate muscle networks and signal synergies was carried out in seven patients with DM1 and ten healthy controls (HC).

**Results:**

Patients with DM1 showed a commingling of low and high frequencies among muscle at both within– and between–limbs level, a weak direct neural coupling concerning inter–limb coordination, a modest network segregation, high integrative network properties, and an impoverishment in the available signal synergies, as compared to HCs. These network abnormalities were independent from muscle weakness and myotonia.

**Conclusions:**

Our results suggest that gait impairment in patients with DM1 depends also on a muscle network deterioration that is secondary to signal synergy deterioration (related to CNS impairment). This suggests that muscle network deterioration may be a primary trait of DM1 rather than a maladaptive mechanism to muscle degeneration. This information may be useful concerning the implementation of proper rehabilitative strategies in patients with DM1. It will be indeed necessary not only addressing muscle weakness but also gait-related muscle connectivity to improve functional ambulation in such patients.

## Background

Myotonic dystrophy type 1 (DM1) is a multi–system disorder involving skeletal muscles and extra–muscular districts, characterized by myotonia, and muscle weakness and atrophy. Patients with DM1 also complain of symptoms belonging to Central Nervous System (CNS) (with minor cognitive deficits, sleep disorders, mild cortical atrophy and white matter abnormalities), endocrine system (e.g. diabetes), osteoarticular system (dysmorphisms), eyes (cataracts), and heart (conduction defects) [1].

DM1 is the most common adult form of muscular dystrophy, and is due to the expansion of CTG–repeat in the non–coding 3′ UTR of the myotonic dystrophy protein kinase gene of > 50 times [1]. The range of CTG repeats determines disease severity, being classified into mild (E1, 51–149 repeats), classic (E2a; 150–450 repeats; and E2b, 451–1000 repeats), and Congenital form (E3, > 1000 repeats) [2, 3].

In the E2 form, patients with DM1 complain of distal and axial muscle weakness, as well as myotonia in both upper and lower limbs [1]. This consequently impairs gait and balance, with an augmented risk of falls, and a negative impact on functional capacities of daily living [4]. A proper rehabilitation program targeting gait and balance is indeed mandatory to procrastinate the use of the wheelchair, to maintain patient’s quality of life, and to maximize patient’s physical and psychosocial functions, even in the late stages of the disease [5–7]. However, only a few data are available on how a proper gait and balance rehabilitation program has to be implemented in patients with DM1.

Also, converging data on gait and balance pathophysiology in DM1 are still missing [5]. Up to date, gait pathophysiology of DM1 has been investigated in small cohorts of patients by using lower–limb segmental kinematics from infrared–emitting diodes, kinematics analysis coupled with ground reaction force measurements and wireless electromyography (EMG) [8–10]. Altogether, these studies suggest that gait and balance impairment may simply depend on the degree of lower–limb distal muscle weakness (with a variable contribution of myotonia). The correlation between postural impairment and gait limitation and between myotonia severity and gait impairment remains instead partially unclear [8–10].

It is worthy to remember that gait and balance impairment in patients with DM1 are of particular nature, as they are unavoidably affected by CNS involvement [11]. This calls into account a potentially significant role of the deterioration of muscle coordination in gait and balance abnormalities in patients with DM1. Muscle synergy is defined as the temporal pattern of co-activations of different muscles recruited by a single neural command signal [12–14]. Indeed, a single muscle can be part of multiple muscle networks, and a single network can activate various muscles [14]. The pathophysiological role of muscle connectivity deterioration in gait abnormalities in DM1 is suggested by the observation that some patients complain of significant alterations in gait and balance despite a mild weakness and myotonia severity [15]. The knowledge of muscle connectivity impairment in patients with DM1 might be important in order to understand: 1) motor behavior adaptations concerning balance and gait; and 2) why recovering abnormal muscle coordination through patient–tailored rehabilitation programs may be helpful to improve functional gait.

Given the particular nature of the motoric deterioration in DM1 (i.e. of both central and peripheral origin), our study was aimed at demonstrating muscle connectivity deterioration in these patients by a CNS perspective, namely signal synergies [16]. About that, we carried out a time-frequency spectral coherence analysis applied on surface EMG data recorded from eight lower limb muscles while walking upright to identify the muscles networks involved in ambulation [17]. This caveat is fundamental given that we aimed at investigating the neural drive to muscles deduced from EMG data, whereas classic muscle synergy studies typically focus on covariations in the temporal domain purely (rather than a time-frequency approach as in our study). More in detail, we used the graph theoretical analyses to quantify the characteristics of the network topology and statistically compare the muscle networks during walking, so to verify the degree of preservation of the patterns of widespread connectivity between muscles that needs to be coordinated to walk (as reflected by the neural drive to muscles deduced from EMG data). Indeed, muscle networks usually show synchronizations at multiple distinct frequency bands, which reflect the neural synchrony involved [17]. Thus, the absence of different muscle networks across distinct frequency bands would suggest the deterioration of CNS networking subtending muscle network organization in DM1 [17]. Whether the postural impairment has a biasing effect on gait limitation in patients with DM1 (regardless of muscle network deterioration) was investigated using an electronic baropodometry. Last, we sought at determining whether the neural drive to muscles was abnormal because of either muscle impairment (i.e., maladaptive muscular networking) or CNS damage (given that DM1 is a multi–system disorder also involving the CNS, and muscle coordination is of neural origin).

## Methods

### Participants

**Fig. 1:**
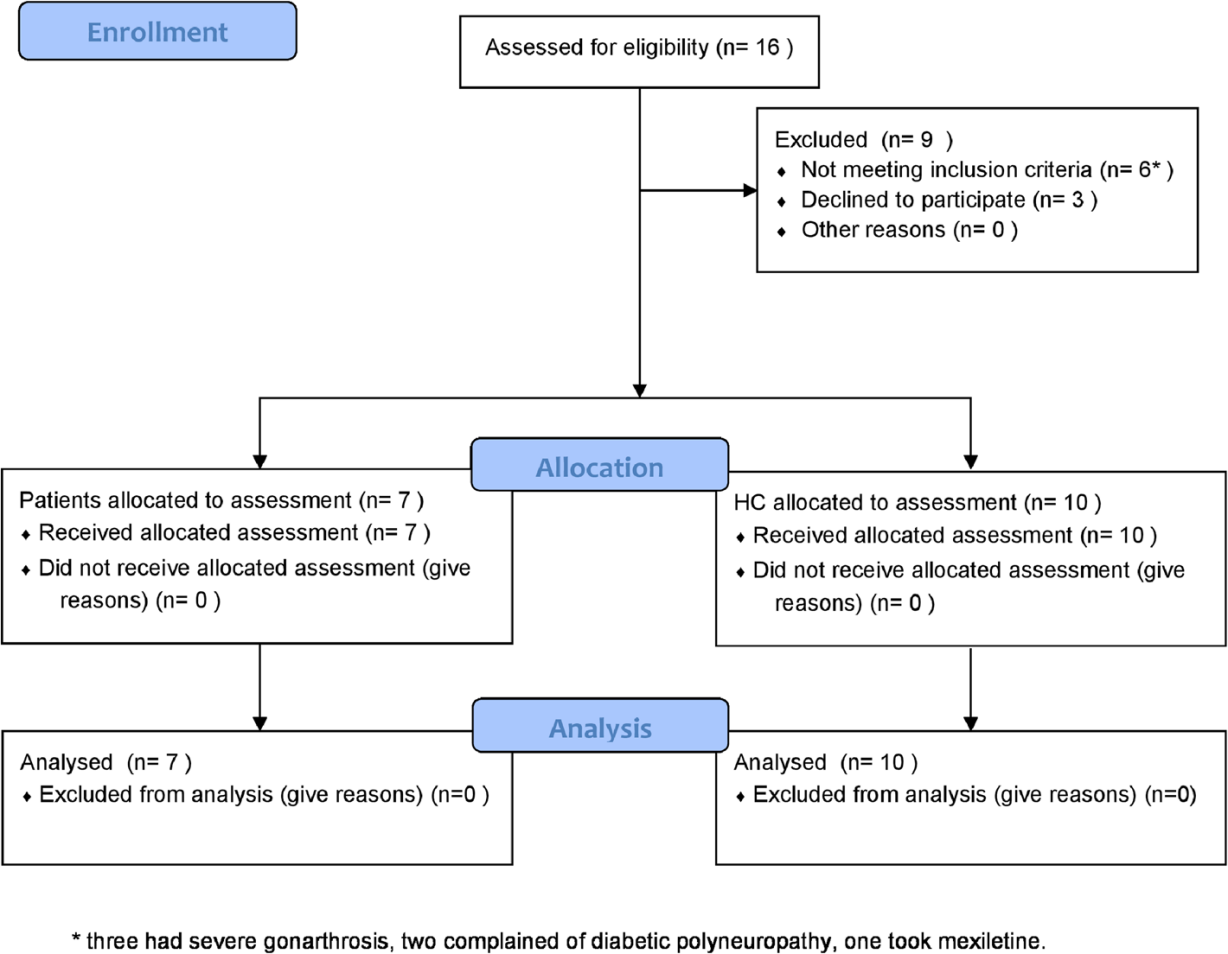
Subjects’ experimental flow

Seven patients with a clinical, genetic, and electrophysiological evidence of DM1 (six females and one male, mean age of 35 ± 18 years) were included in the study between January and October 2018. The inclusion criteria were as it follows: no clinical or laboratory evidence of other neurological disorders or diseases affecting the peripheral nervous system; no orthopedic illness potentially interfering with ambulation; no intake of drugs modifying muscles and nerve excitability. Clinical–demographic characteristics are reported in Table 1. Ten normal individuals were enrolled as control group (HC) (four males and six females, mean age of 38 ± 5 years). The study was reviewed and approved by the Ethics Committee of our institute (ID: IRCCSME#41/2017), and conducted according to the principles expressed in the Declaration of Helsinki. All subjects gave their written informed consent to study participation. Subjects’ experimental flow is summarized in Fig. 1.

**Table 1:** DM1 clinical–demographic characteristics

gender, age	CTG expansion^a^	DM1 onset	Comorbidities	CRS	MSS
F, 49y	E2	25y	headache	2	2
F, 23y	E2	16y	nasal turbinate stenosis	2	2
M, 19y	E2	17y	–	1	3
F, 45y	E2	22y	BS1, OSAS	2	3
F, 29y	E2	15y	headache	2	2
F, 17y	E2	15y	–	1	3
F, 65y	E2	30y	headache, HBV–hepatitis, slight respiratory failure	2	2

Legend: CRS (Clinical rating scale) for DM1 (Dystrophic Myotonia type 1): 1 = presence of myotonia and/or mild functional weakness without functional impairment; 2 = moderate muscle weakness leading to some degree of functional impairment; 3 = muscle weakness with severe functional impairment and in some cases resulting in the subjects being bound to a wheelchair; 4 = bedridden. MSS (myotonia severity scale) for DM1 from 0 (absence of myotonia) to 4 (the worst myotonia experienced). BS1 Brugada syndrome type 1.OSAS Obstructive Sleep Apnea Syndrome. ^a^ Range of CTG expansion: E1: 20–150; E2: 150–1000; E3: > 1000

### Gait analysis

Surface myoelectric signals were sampled at 1 kHz and filtered at 5–400 Hz using the Smart Analyzer (Version 1.10.469.0; BTS, Milan, Italy) from rectus femoris (RF), biceps femoris (BF), tibialis anterior (TA), and gastrocnemius medialis (G) of both lower limbs. After careful preparation of the skin, the bipolar adhesive surface electrodes were placed over the muscle belly in the direction of the muscle fibers according to the European recommendations for surface electromyography (SENIAM) [18, 19]. An inter–electrode distance of at least 2 cm was used to minimize cross-talk between EMG signals [20]. Thus, signals were analyzed for root–mean–square (RMS) (a temporal parameter estimating muscle activation) to investigate lower–limb muscle activation during gait. The different phases of gait cycle were signaled by an accelerometer (G-Sensor) (BTS, Milan, Italy) fixed to the lumbar level by a Velcro strap. Gait data were recorded while the participant walked along a 10–meter walkway at their usual walking speed. Gait data were collected twice. Participants wore tight fitted clothing and were barefoot. The BTS software furnishes a measure called gait quality index (GQI). This is an overall gait performance score reflecting the quality of gait (https://www.btsbioengineering.com/products/freeemg-surface-emg-semg/), where normal gait is reflected by a 60:40% stance:swing ratio (SSR) (i.e., ratio between stance from heel strike to toe–off, and swing phase duration from toe–off to heel strike), and a normal step cadence (about 1.9 Hz) and gait cycle duration (GCD) (about 1.1 s from one right heel strike –namely, the initial contact– to the next one –namely, end of terminal swing). GQI values were averaged from the two runs and used for subsequent analyses. The higher the GQI was, the better the gait performance resulted.

### Stance analysis

For the baropodometric evaluation, all participants were instructed to maintain an upright standing bipodalic position on a force plate of the electronic baropodometer (EPS R-1-KINETEC®; RO + TEN, Arcore, Italy), with the feet wide apart at 30 degrees, both arms at their sides, and looking straight ahead to a 3-m distant achromatic target. Participants had to stand as still as possible for 2 min. The task was repeated twice. We measured: 1) the ellipse sway area, which was calculated based on the 90% of oscillations of center of pressure (COP) amplitude along the main axes of the ellipse (anterior–posterior and medial–lateral); the ellipse sway area was also calculated with the eyes closed to compute the Romberg quotient. 2) The COP path length was defined as the real displacement of the center of pressure in reference to the plantar support, i.e., of the center of the distribution of the total force applied to the supporting surface). 3) The Romberg quotient (ratio of ellipse sway area with eyes closed/open). 4) The stabilometric index, which is automatically calculated by the device, reflects the distribution of the vertical forces on the support plane and the stability of, and the energy used by, the subject to precisely maintain postural control [21]. 5) The static index, which is also automatically calculated by the device, reflects the average position of the center of gravity of the body and its standard deviation [21].

### Muscle connectivity analysis

EMG data processing preceding signal synergy extraction consisted of six sequential steps [17].

1. the EMG signals recorded from the eight surface EMG sensors were band-pass re filtered (0.5–70 Hz), down-sampled to 200 Hz, and rectified using Hilbert transform.
2. we extracted the power spectral density (PSD) (to investigate the spectral content of the EMG envelopes), which was individually normalized by the spectral resolution employed to digitize the signal, so to investigate the spectral content of the EMG envelopes (2 Hz bandwidth, thus obtaining 35 bins).
3. we estimated the intermuscular coherence (IMCoh) of the EMG envelopes to map undirected functional muscle networks (using Welch method with window length of 1 s and an overlap of 750 μs, averaging and squaring the values across the two trials to obtain magnitude–squared coherence) [22]. Coherence is commonly used to investigate the linear coupling between neural activities [23]. Specifically, the IMCoh estimated the descending output to different muscle groups between all pairs of muscles tested during walking at self–paced speed, so to define the edges of the undirected muscle network [24].
4. we calculated the partial directed coherence (PDC) (a measure of network connectivity) to map the directed muscle networks accounting for the temporal flow of information among muscles. To this end, we used the coefficients of a multivariate autoregressive model [25]. We had to estimate both undirected and directed graph as they reflect different network properties. The former graph (undirected network) considers a set of objects (vertices or nodes or points or actors) that are connected together with bidirectional edges (links or lines or ties). The latter (directed network or diagraphs) consider nodes where the edges (arcs) point in a direction. Thus, the undirected network reflects the functional associations between nodes (namely, muscles), whereas the directed network refers to the node-wise direction of information flow [26, 27]. We also had to calculate PDC because IMCoh could be biased by common inputs arising from other structures (indirect paths), thus overestimating the strength of direct connections between motor–unit pools innervating different muscles [28]. To do that, we individually defined the optimal model order using Akaike’s information criterion. Then, we computed the magnitude–squared Coh from the model coefficients to compare the model with the Coh spectra directly estimated from the data, in order to validate the model.
5. the network topology was studied using both of the undirected (IMCoh data) and directed (PDC data) muscle network connectivity measures in distinct frequency components (and their corresponding coupling strength by applying a non-negative matrix factorization -NMF). Frequency decomposition into non–negative factors was obtained using an alternating least squares algorithm applied to 2 Hz bins. Thus, we obtained two nonnegative matrices for each frequency bin reflecting one the pair-wise muscle spectral signatures (basis vectors), the other the pair-wise muscle connectivity strength.
6. these last data were used to calculate the network measures clustering coefficient (CC), global efficiency (GE), and betweenness–centrality (BC). The CC is a measure of functional segregation and is equivalent to the fraction of the node’s neighbors that are also neighbors of each other [29]. The mean CC for the network reflects, on average, the prevalence of clustered connectivity around individual nodes [30], with higher values indicating a more functionally segregated network. The average shortest path length between all pairs of nodes in the network is known as the characteristic path length of the network, which is the most commonly used measure of functional integration [29]. The average inverse shortest path length is a related measure known as the GE. Higher values of GE indicate a more functionally integrated network. Paths length can be generalized for directed and weighted networks: weighted path length is equal to the total sum of individual link lengths, which are inversely related to edge weights [30]. BC is a measure of centrality used to identify hubs in a network. It is defined as the fraction of all shortest paths in the network, which pass through a given node. BC is computed equivalently on weighted and directed networks, if path lengths are computed on respective weighted or directed paths.

### Signals synergy analysis

Classically, synergies can be deduced from EMG data using factor analyses like NMF or principal component analysis [31–34]. We estimated synergies from EMG data using an NMF method based on the Lee–Seung algorithm [33], which is a basic and fast NMF algorithm with multiplicative updating rules.

EMG data from each trial (gait cycle) were used as input to the NMF algorithm. Muscle activity signals M were arranged to form an *m × n* matrix, where *m* is the number of measured muscles and *n* the number of samples. Thus, M_*m × n*_ data was subjected to NMF algorithm (i.e., they were factorized using the Lee–Seung algorithm) to obtain the synergy matrix W and the synergy recruitment matrix C according to the formula M_*m × n*_ = (W_*mk*_ × C_*nk*_) + E_*m × n*_, where *k* is the number of synergies and E the residuals. The number of synergies needed to reconstruct the original muscle activity data during gait was calculated on the variability accounted for (VAF) the values of M_*m × n*_ and E_*m × n*_. When VAF was higher than the 90% threshold, then the number of synergies was deemed sufficient to regard the factorization results W_*mk*_ and C_*nk*_ as representative of the data set M_*m × n*_.

### Statistical analysis

Descriptive statistics were presented for all outcomes for both groups. Kolmogorov–Smirnov test estimated the normality of the distribution of data (all data were normally distributed, *p* > 0.1). One–way analysis of variance (ANOVA) (factor group: two levels) was used to compare static and gait parameter between patients with DM1 and HC with *post–hoc* comparison between groups (Bonferroni correction for multiple comparisons).

When comparing muscle connectivity data, ANOVA was added with the factors *muscle pair* (56 levels) and *frequency bin* (35 levels) were implemented in the ANOVA, beyond the factor *group*. Significance was set at *p* < 0.05 for all tests. Bonferroni correction for multiple comparison was applied in *post-hoc t-*tests.

Pairwise correlations between all tested variables were assessed using Spearman’s rank correlation coefficient. A multivariate regression analysis was used to determine whether and which factor(s) among muscle connectivity data influenced signal synergy. The statistical analyses were conducted using the Stat-View® software program (Hulinks Inc.; Tokyo, Japan).

## Results

### Stance and gait data

Gait was abnormal in all patients concerning the measures we evaluated. Patients showed a slightly longer gait cycle duration, as compared to HC (1.4 ± 0.7 vs. 1.1 ± 0.9 s; F _(1, 15)_=4, *p* = 0.04 η^2^ = 0.2), lower GQI (81 ± 5 vs. 98 ± 2%; F _(1, 15)_=8.1, *p* = 0.009, η^2^ = 0.35), and lower step cadence (1.4 ± 0.7 vs. 1.9 ± 0.1 Hz; F _(1, 15)_=113, *p* < 0.001, η^2^ = 0.88), whereas there were no group differences concerning SSR (1.5 ± 0.5 vs. 1.6 ± 0.5; F _(1, 15)_=3, *p* = 0.09, η^2^ = 0.16). Further, patients walked at a slower pace than HC did (0.6 ± 0.04 vs. 1.2 ± 0.01 m/s; F _(1, 15)_=72, *p* < 0.001, η^2^ = 0.83).

Balance presented clear abnormalities in patients with DM1, as compared to HC. Patients showed greater COP path length (556 ± 40 vs. 370 ± 57 mm; F _(1, 15)_=102, *p* < 0.001, η^2^ = 0.87), ellipse sway area (163 ± 30 vs. 125 ± 39 mm^2^; F _(1, 15)_=24, *p* = 0.001, η^2^ = 0.57), stabilometric index (21 ± 3.5 vs. 8 ± 1.5; F _(1, 15)_=17, *p* = 0.002, η^2^ = 0.48), and static index (9 ± 1.2 vs. 5 ± 1.5; F _(1, 15)_=19, p = 0.001, η^2^ = 0.55). There were no group differences concerning Romberg quotient (0.9 ± 0.2 vs. 1.1 ± 0.2, *p* = 0.2).

### Muscle connectivity data

**Fig. 2:**
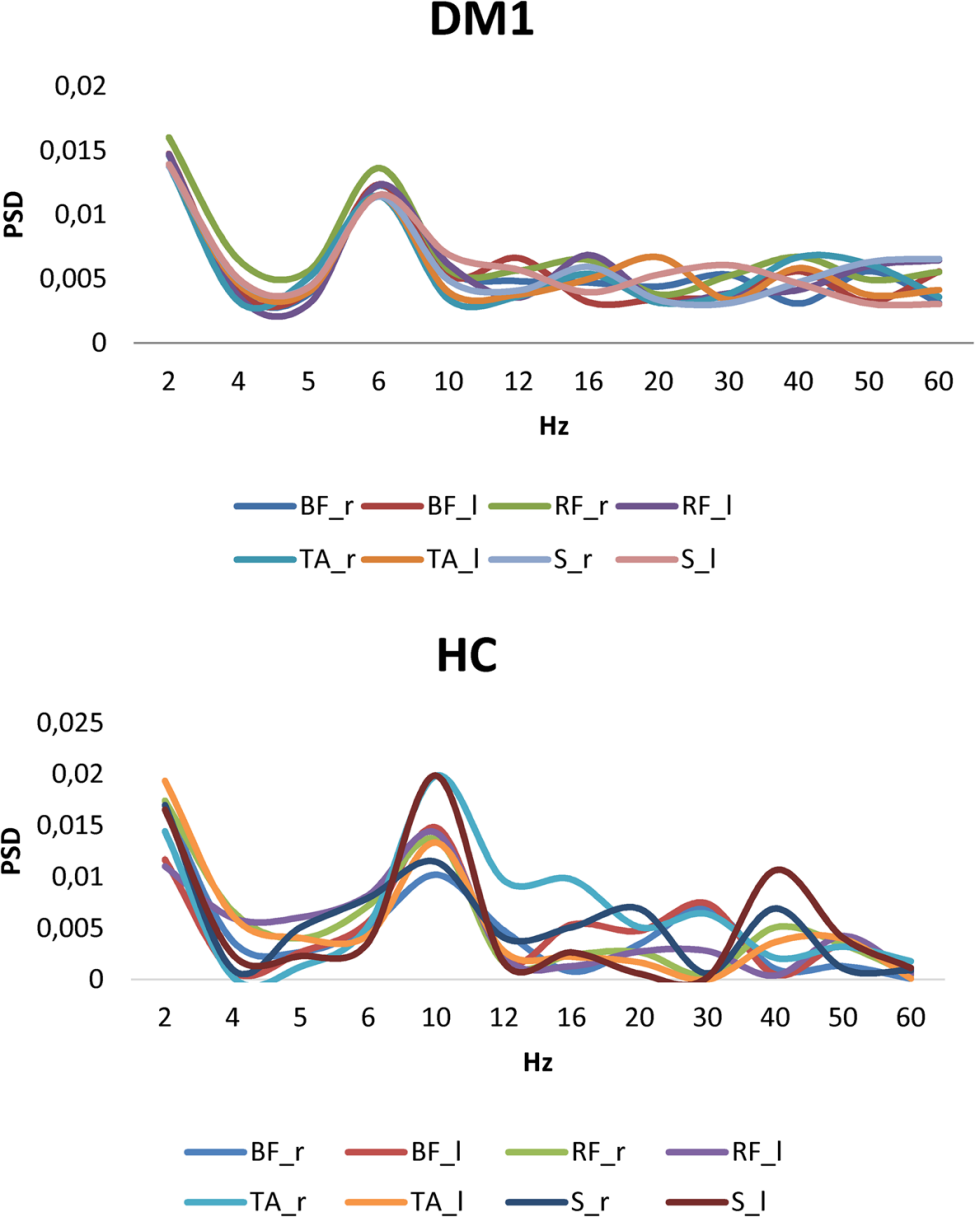
Normalized power spectral density (PSD) of the EMG envelope (resulting from power spectra averaged across homologous muscles and subjects and normalized to total power) for different muscles (GM gastrocnemius, TA tibialis anterior, RF rectus femoris, BF biceps femoris) and frequencies in patients with Dystrophic Myotonia type 1 (DM1) and healthy controls (HC)

Both the groups showed similar PSD values across muscles (*group×muscle* interaction *p* = 0.9), but there was a significant difference concerning frequency bins. In fact, patients showed a small frequency peak around 6 Hz, whereas HC peaked around 10 Hz *(group×frequency* interaction F_(34,510)_ = 64, *p* < 0.001, η^2^ = 0.65) (Fig. 2).

**Fig. 3:**
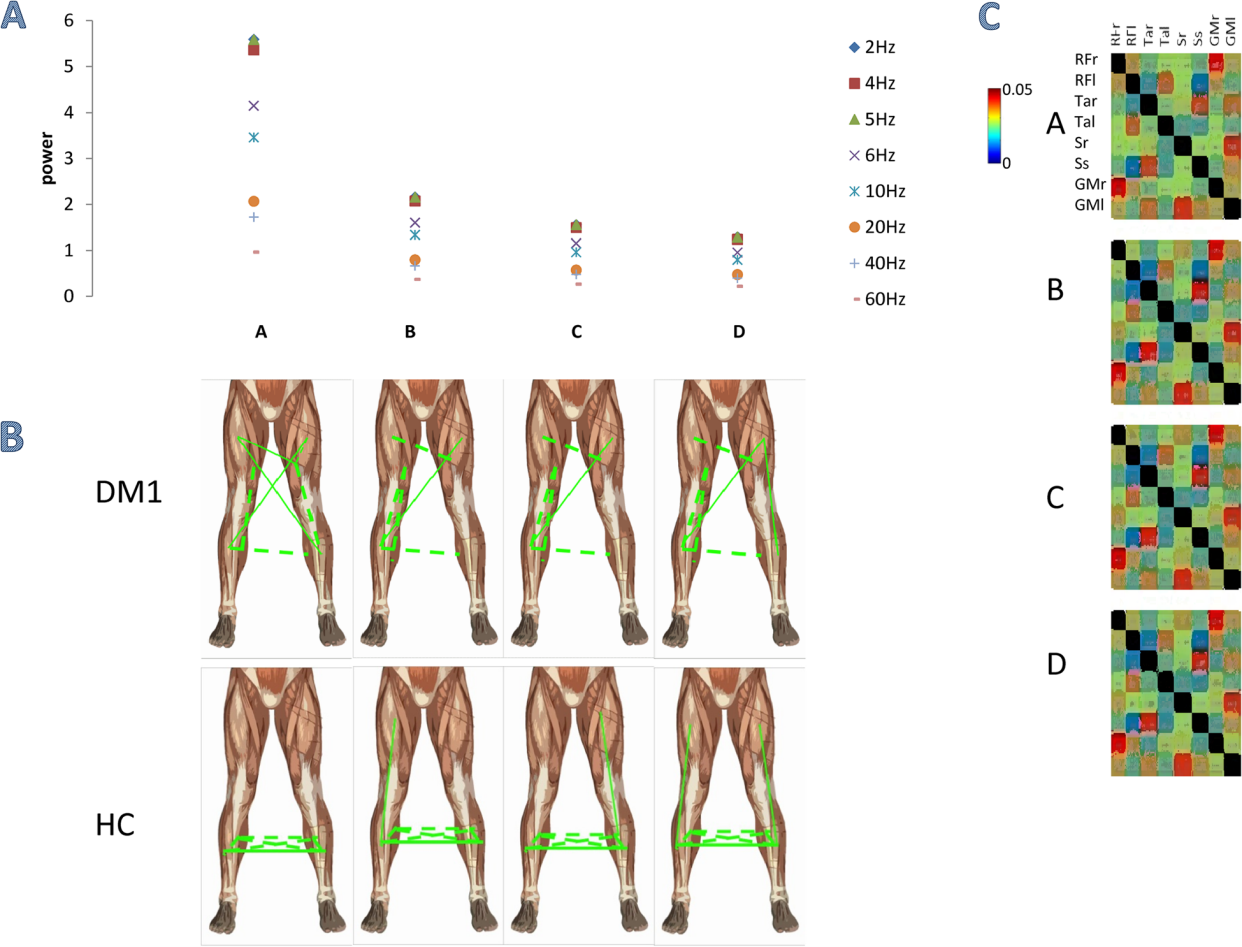
Undirected muscle networks obtained using non–negative matrix factorization of IMCoh. The figure illustrates (**A**) the frequency spectrum of the four identified components (**A–D**); (**B**) the binarized networks obtained using proportional thresholding for the networks corresponding to the frequency components in panel A (the strength of connection is indicated by the width of the lines, continuous among muscles of anterior thigh and leg, dotted for the other muscles); and (**C**) detailed significance in the correlation matrix

The groups showed different IMCoh values across muscle pairs and frequency bins (*group×muscle-pair×frequency-bin* main interaction F_(1870,28050)_ = 14, p < 0.001, η^2^ = 0.48). In particular, four main frequency components emerged after the decomposition of the EMG frequency spectra (Fig. 3). A component peaked around 5 Hz and indicated high Coh between proximal and distal of both sides. The second (5–12 Hz), third (12–25 Hz), and fourth component (25–45 Hz) substantially involved the same muscle pairs. Thus, we found a gradual transition from a high bilateral IMCoh between lower leg muscles for the lowest frequency component observed in HC to a strong IMCoh with upper–lower leg muscles for the highest frequency components, in parallel to a reduced inter–limb IMCoh (Fig. 3). In particular, IMCoh in patients was stronger for proximal–distal muscle pairs within the same leg, and proximal-proximal and distal-distal muscle pairs of both limbs (RF_right-S_left *p* = 0.02, RF_right-S_right *p* = 0.01, RF_right-TA_left p = 0.01, and RF_right-TA_right p = 0.01). On the contrary, IMCoh was high only concerning RF–S and TA–S of both lower limbs, both TA and both RF (all *p* < 0.001). HC showed instead a strong IMCoh limitedly to TA–S of both limbs and VL–TA in each side. Each between–group comparison for each muscle pair and component was significant (*p* < 0.001).

**Fig. 4:**
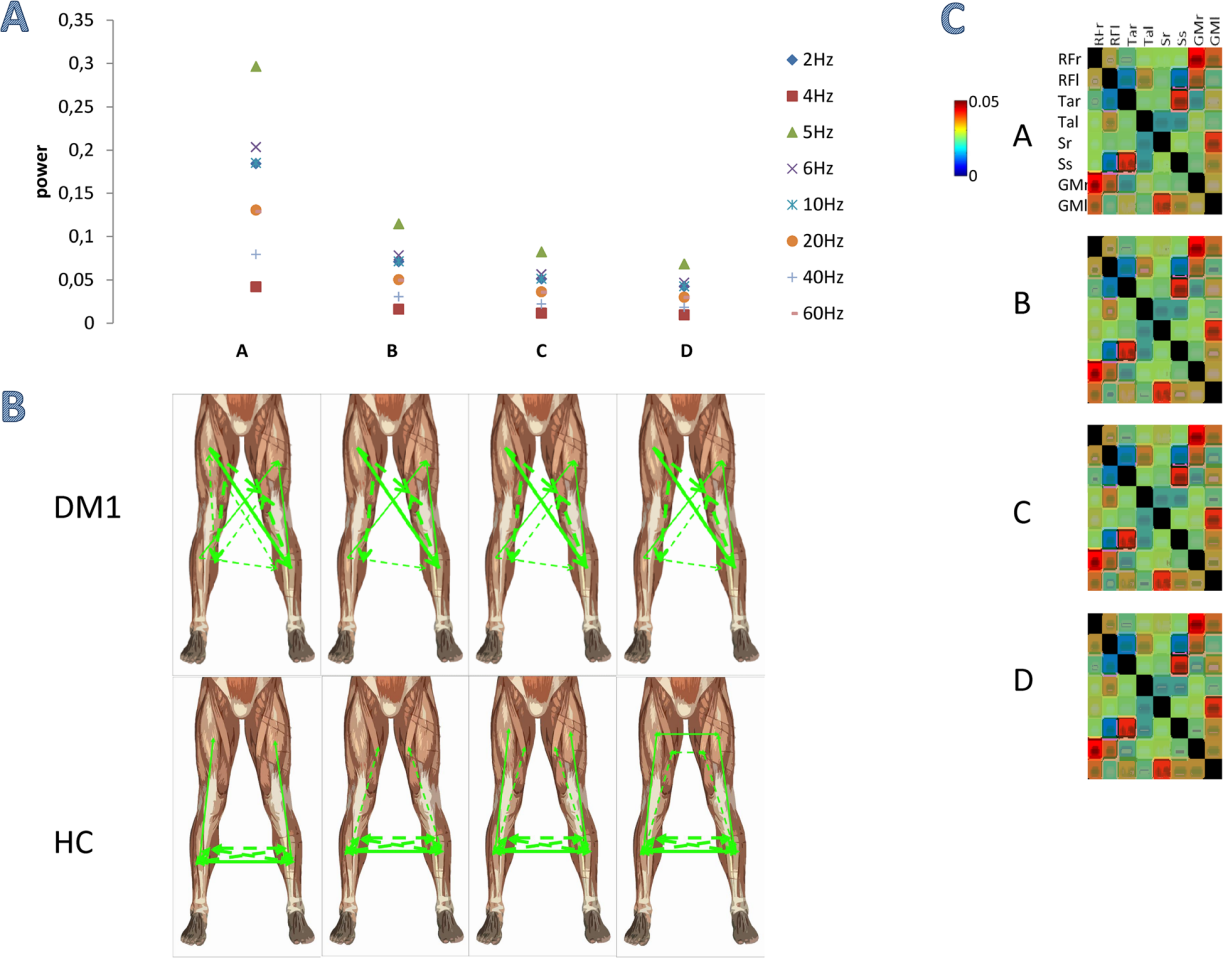
Directed muscle networks obtained using non–negative matrix factorization of the PDC. The figure illustrates (**A**) the frequency spectrum of the four identified components (**A–D**); (**B**) the binarized networks obtained using proportional thresholding for the networks corresponding to the frequency components in panel A (the strength of connection is indicated by the width of the lines -continuous among muscles of anterior thigh and leg, dotted for the other muscles-, the direction of connectivity by the arrow); and (**C**) detailed significance in the correlation matrix

The groups showed different PDC values across muscle pairs and frequency bins (*group×muscle-pair×frequency-bin* main interaction F_(1870,28050)_ = 9, *p* < 0.001, η^2^ = 0.37). We identified four main directed components (Fig. 4) that were wider (i.e., involved more muscle pairs) than the undirected components (Fig. 3). This suggests the presence of different, strong pairwise connections in each of the components. Overall, PDC had lower values than IMCoh. This depends on the fact that PDC is less affected by inputs from other structures. HC showed instead a directed muscle network pattern characterized by high connectivity between the distal muscles within and between legs (Fig. 4). Each between–group comparison was significant for each muscle pair and component (*p* < 0.001).

**Fig. 5:**
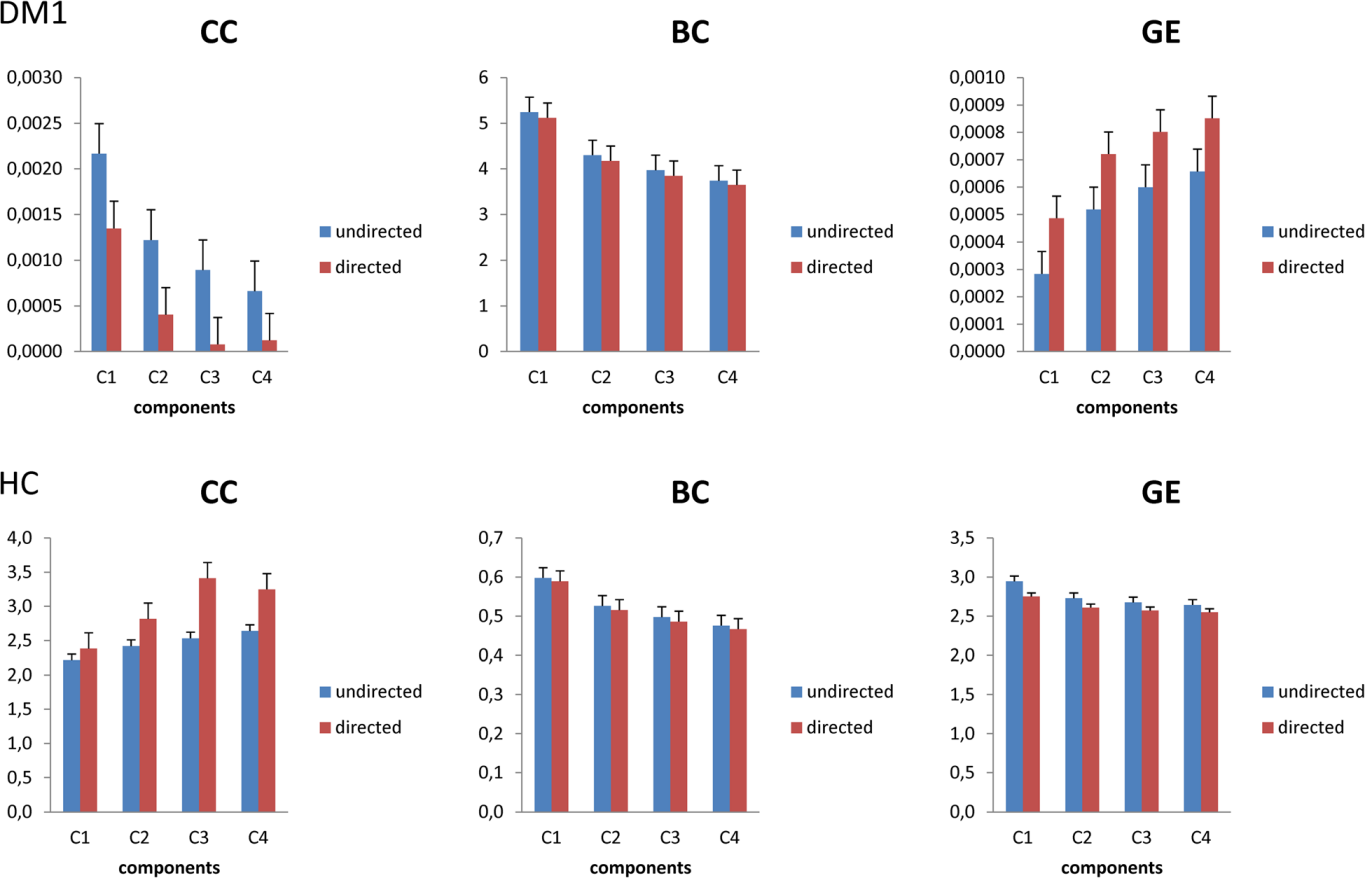
The network metrics used to compare muscle networks across frequencies (the four identified components) were computed based on the weighted connectivity matrices muscle networks, and were thresholded only for visualization purposes clustering coefficient (CC), global efficiency (GE), and betweenness–centrality (BC) were assessed for both undirected (from IMCoh) and directed networks (from PDC). Error bars indicate standard error

Complex network analysis showed a trend to network integration (as reflected by GE and BC values) to the detriment of network segregation (as reflected by CC values) (Fig. 5). In detail, the CC was lower than BC (*p* < 0.001) and GE (*p* < 0.001), with particular regard to the first than the other components in the undirected network (*p* < 0.001) (Fig. 5). Within GE, the last component was higher than the other ones (*p* = 0.01) (Fig. 5). This pattern was totally reversed in the HC, i.e., a higher CC than BC (p < 0.001) and GE (*p* < 0.001). Within each graph measure, the last component was greater than the others in the undirected network (*p* < 0.001), whereas both BC and GE showed non–significant differences. For the directed networks, the trend of the network measures was the same observed in the undirected networks, but there were no significant pair-wise differences between the components in both DM1 and HC group (Fig. 5).

### Signal synergies data

**Fig. 6:**
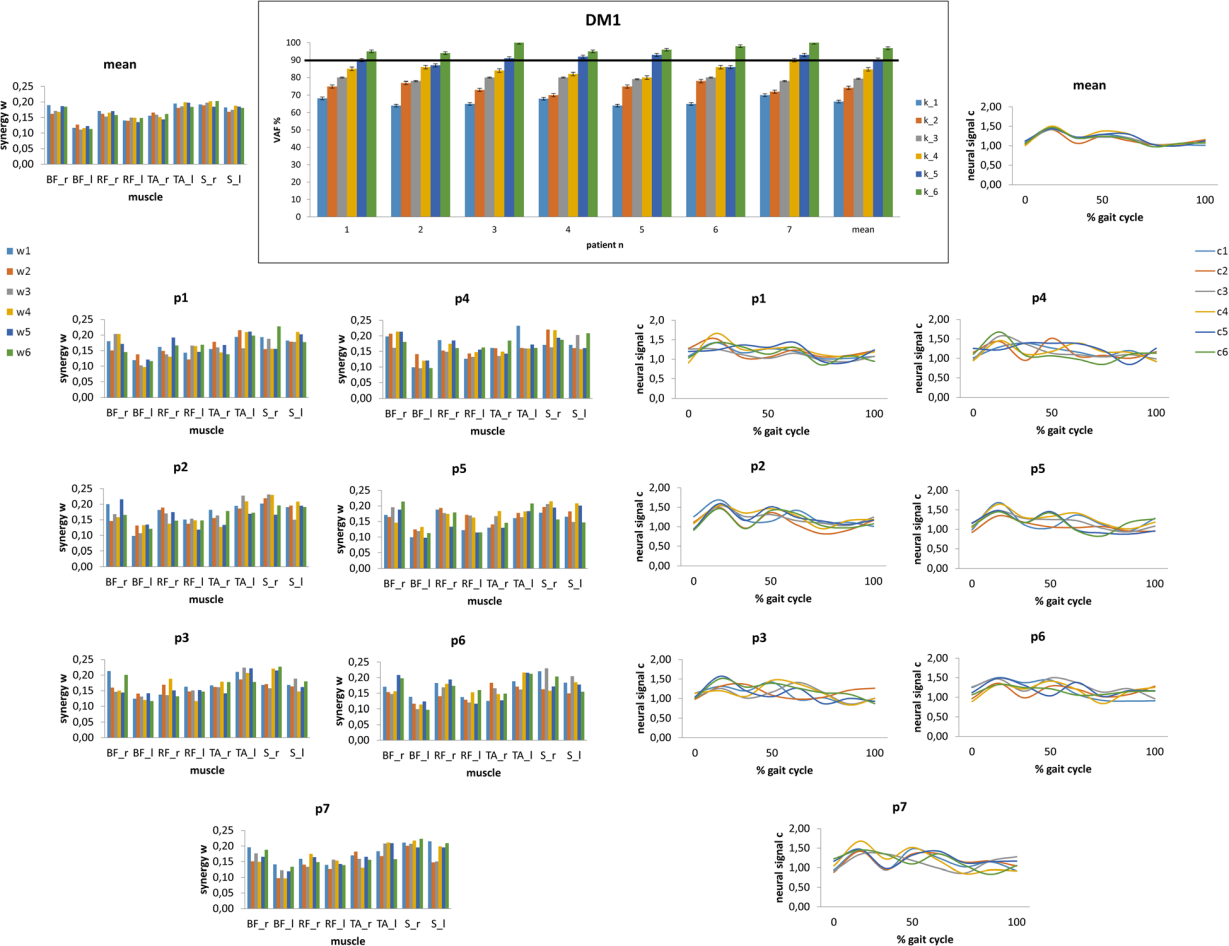
The main top graph illustrates the synergies extracted in each healthy control (and the grand mean) using non-negative matrix factorization. The number of synergies k depended on whether VAF was greater than the 90% threshold (horizontal line). Error bars denote standard deviation. The smaller graphs refer to individual and mean values of synergies W (bar graphs) and synergy recruitments C (time plots)

**Fig. 7:**
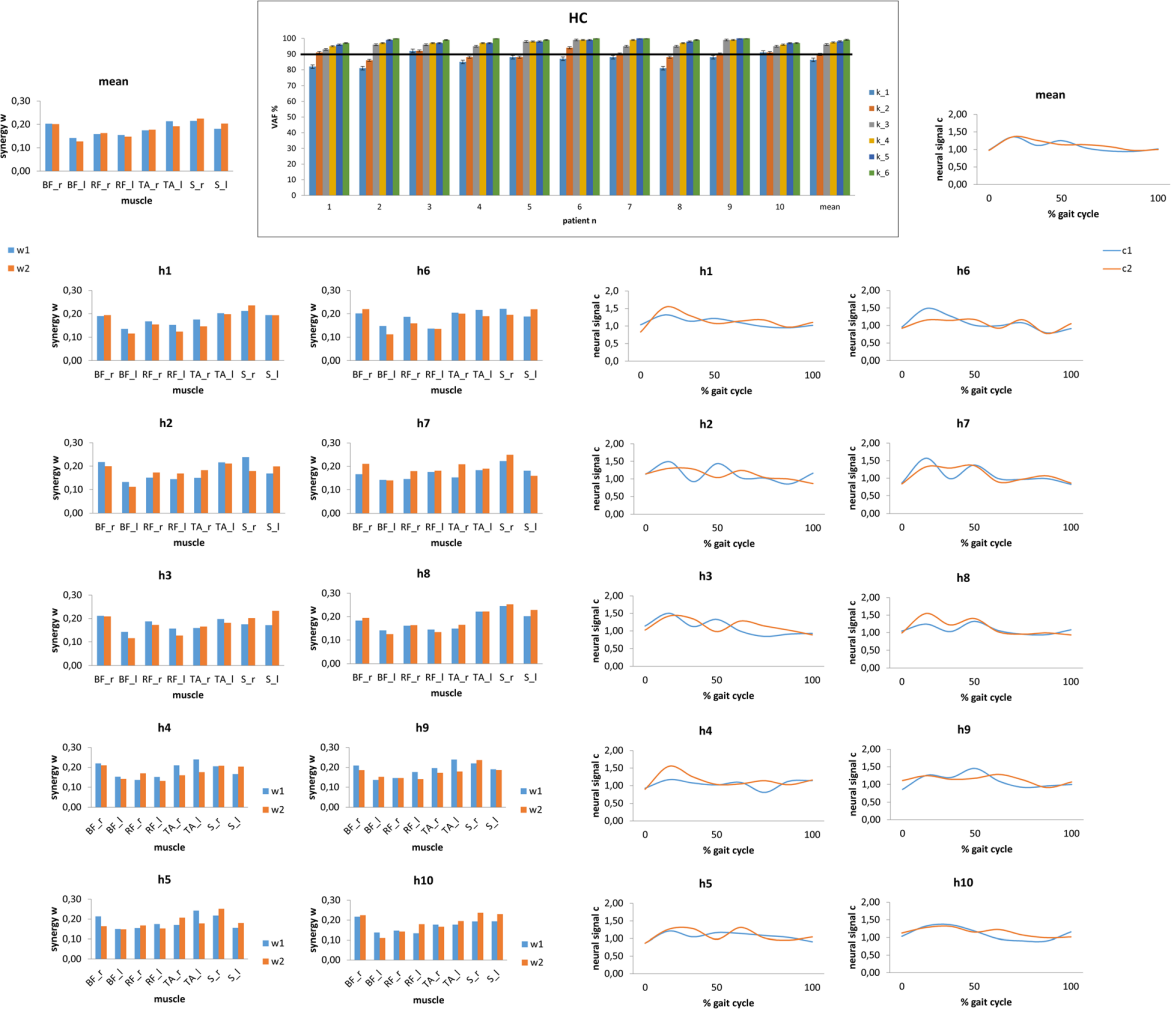
The synergies extracted in each patient with DM1 (and the grand mean), the individual and mean values of synergies W (bar graphs) and synergy recruitments C (time plots)

VAF calculation indicated that one to two synergies *k* were needed to reconstruct the original muscle activity data during gait of all measured muscles in HCs. Thus, we extracted two synergies across participants to facilitate comparison (Fig. 6). Conversely, five to six synergies were required in patients with DM1 (Fig. 7). Patients with DM1 showed that muscle activation varied from a gait cycle to another as compared to HCs (higher C values, *p* < 0.001) (Figs. 6 and 7). In addition, HC used the same synergies across gait cycles (involving S and TA), whereas patients used different synergies from a gait cycle to another (involving BF_right, RF_right and TA_left or RF_left, both TA, and both S), as reflected by the higher W values in HCs (*p* < 0.001) (Figs. 6 and 7).

### Pair-wise correlations

Only the most significant gait and balance features were subjected to correlation analysis with respect to muscle connectivity and signal synergy data. Spearman’s rank correlation test indicated a significant correlation between muscle weakness and stability index (r = − 0.724, *p* = 0.002) and GQI (r = − 0.621, *p* = 0.01). No correlations were found between muscle weakness and any of the muscle connectivity and synergy data. Among these, overall PDC values across muscle pairs and frequency bins significantly affected the overall synergy variance during gait (R^2^ = 0.895, F_(1870,28050)_ = 43, *p* < 0.001, η^2^ = 0.74).

## Discussion

Muscle weakness clearly affects gait in patients with neuromuscular disorders of non-neural origin (e.g., muscular dystrophy) as suggested by previous studies on gait analysis in patients with myopathy [8, 9, 35, 36]. As expected, both balance and gait features were largely abnormal in patients and were significantly related to muscle weakness [37].

Whether muscle weakness directly contributes to muscle network deterioration or muscle network deterioration simply reflects neural impairments remains instead to be clarified. A previous study found that muscle weakness and muscle coordination are not correlated in muscle dystrophy [38]. However, gait and balance impairment in patients with DM1 may have peculiar aspects owing to the involvement of the CNS [39–41], thus potentially biasing muscle coordination. To this end, we employed a time-frequency approach on EMG data analysis rather than focusing on covariations in the temporal domain, so to gain information on signal rather than muscle synergies. To the best of our knowledge, this is the first attempt to study the CNS networking subtending muscle networks during walking upright in patients with DM1.

Muscle connectivity analysis offered three new data concerning a causative correlation between gait and balance impairment and the deterioration of muscle networking (i.e., beyond muscle weakness on its own).

1. Patients with DM1 show a deterioration of motor network organization as suggested by the IMCoh and PDC patterns. The former reflects muscle indirect connectivity (i.e., the statistical association between muscle activities), the latter the direct connectivity (i.e., the direction and the temporal flow of information within muscle pairs) [16, 42]. In particular, undirected connectivity maps revealed that patients complained of a commingling of low and high frequency ranges within both short- and long–range connections. In other words, functional muscle connectivity was observed at multiple distinct frequencies and, conversely, multiple connections between different muscle pairs were observed at each frequency. Hence, patients missed the functional connectivity patterns that occur normally to simplify and make more efficient integration processes (i.e., low frequencies within long–range connections and high frequencies within short–range connections). This denotes a detrimental signal integration at different temporal and spatial scales, which could mirror what would occur at cortical network level owing to CNS involvement in DM1 [23, 43]. About that, it has been shown that peculiar patterns of fronto-parietal and cerebellum-cerebrum disconnection are detectable in patients with DM1, despite a global preservation of the brain topological properties [11, 44–46]. The evaluation of undirected connectivity maps was carried out by using PDC as a measure of network connectivity taking into account only the common input from other areas (thus being a more direct reflection of coupling between two areas) [25]. It revealed a loss of information flow directionality, i.e., there were multiple, bidirectional connections within and between muscle at both within- and between-leg level in patients with DM1. This directed network pathology may reflect a loss of the topological hierarchy at the level of M1 and, probably, premotor-M1 connectivity, which are both important for muscle coordination [47, 48]. Noteworthy, no correlation existed between muscle weakness and muscle connectivity (both directed and undirected) deterioration.
2. Complex network analysis revealed a greater network integration to the detriment of network segregation, thus suggesting a reduced capacity of DM1 brain to segregate information into distinct modules. This may result in a signal synergy deterioration [47–49]. In fact, such a network deterioration suggests the presence of disorganized top-down projections from premotor to motor cortical networks with, consequently, a few common synaptic output to spinal networks (or central pattern generators) [50–53]. The distortion in top-down projections is also suggested by the recently reported abnormalities in corticospinal excitability and sensorimotor plasticity [54–58].
3. Patients showed an impoverishment in motor repertoire in terms of available synergies, i.e., more synergies were required to walk in patients as compared to HCs. In addition, a greater gait cycle to gait cycle synergy variability and a more variable number of synergies to be employed as well as of muscle to be activated were appreciable in patients with DM1, as compared to HCs. These data suggest that patients with DM1 suffered from a decreased complexity of motor control during gait (as pointed out by the high total variance accounted for by one synergy and by complex-network data analysis). Moreover, the variability in the muscle required to walk indicated that the ankle joint strategy (as reflected by S and TA involvement) was sufficient to gait in HCs, whereas patients with DM1 had to harness both hip and ankle joint strategies in gait to compensate for synergy variability across gait cycles. Notably, no correlation existed between muscle weakness and muscular networking deterioration.

Altogether, these three findings suggest that gait and balance impairment in patients with DM1 may depend on the deterioration of the neuromuscular networks supporting muscle coordination, beyond muscle weakness. In fact, the correlation analyses suggested that the data of signal synergy and muscle connectivity were significantly correlated. Further, both the detrimental muscle connectivity and the signal synergy impairment were unrelated to muscle weakness. These data also substantiate the reason why to recover muscle coordination through patient–tailored rehabilitation programs may be helpful to improve functional gait. It will be indeed necessary not only addressing muscle weakness but also gait-related muscle connectivity to improve functional gait in such patients. However, larger-sample studies are necessary to confirm this statement.

Interestingly, our data suggest that muscle network impairment may depend primarily on a failure of the subtending CNS networks. At least two evidences may support this issue.

1. a disorganized muscle network pattern, in parallel to the impoverishment of available signal synergies during gait, leans more to a primary than maladaptive muscle network organization. The topological indices of CNS connectivity are reported as relatively preserved within and upstream the motor networks, thus suggesting the activation of compensatory, albeit maladaptive, mechanisms of brain plasticity to contrast muscular impairment [44]. We instead found a commingling of the patterns of muscle coordination that occur to adapt to or to recovery from brain damage (e.g., stroke), including preservation, merging, and fractionation of signal synergies [59]. Specificaaly, the abnormalities in IMCoh, PDC, and network topology suggest that motor networks are capable to generate only weak neural coupling from premotor to motor areas, with a consequential signal distortion and, eventually, muscle network fragmentation and disorganization.
2. we did not find any correlation between muscle weakness and coordination, so that neuromuscular networking impairment is likely not to depend on peripheral factors.

### Limitations and conclusions

Given the small number of enrolled patients, our data are not easily generalizable, taking also into account that patients with DM1 can be significantly heterogeneous even within the same family. Thus, larger samples are required to confirm our promising data. In particular, it would be important to stratify network analysis by age and disease duration, as the progressivity of DM1 can affect gait parameters even year by year. A putative contribute of spinal network should not be neglected. In fact, IMCoh and synergy organization depends also on spinal networks and central pattern generators, which may be affected either primarily or secondarily to CNS and/or muscle impairment [52, 53].

One may observe that muscle connectivity data in both directed and undirected networks and signal synergies were common across patients despite their heterogeneity. Indeed, a certain degree of consistency in brain connectivity abnormalities (that we hypothesized to be reflected by signal synergies) has been shown among patients with DM1, despite their clinical heterogeneity [11]. We may postulate that the balance between the loss of connectivity within motor and premotor areas and compensatory mechanisms in some others (e.g., the cerebellum) might explain the mismatch between brain connectivity impairment and the motor and non-motor symptomatological scenario observed in these patients [11, 44]. We may also speculate that such a conservative connectivity impairment among patients suggests more a primary trait of the disease rather that a compensative or maladaptive process to muscle dystrophy.

Anyway, our work suggests that gait and balance impairment also depend on specific CNS mechanisms that are primarily pathologic, rather than maladaptive to muscle deterioration. This information may be useful concerning the implementation of proper rehabilitative strategies in patients with DM1. It will be indeed necessary not only addressing muscle weakness but also muscle coordination during gait to improve functional gait in such patients. About that, plasticity–targeting neurorehabilitative interventions could be required. Last, the electrophysiological assessment we performed may be useful to more objectively: 1) determine the patients who may benefit from a specific intervention; 2) track gains in motor functions; 3) compare treatment strategies; and 4) tailor the treatment to the specific patient’s needs.

## Data Availability

Please contact the corresponding author for data requests.

## Abbreviations

ANOVA: Analysis of variance
BE: Betweenness–centrality
BF: Biceps femoris
CC: Clustering coefficient
CNS: Central Nervous System
COP: Center of pressure
DM1: Myotonic dystrophy type 1
EMG: Electromyography
G: Gastrocnemius medialis
GCD: Gait cycle duration
GE: Global efficiency
GQI: Gait quality index
HC: Healthy controls
IMCoh: Intermuscular coherence
NMF: Non-negative matrix factorization
PDC: Partial directed coherence
PSD: Power spectral density
RF: Rectus femoris
SSR: Stance:swing ratio
TA: Tibialis anterior
VAF: Variability accounted for
